# Hypothermia inhibits the propagation of acute ischemic injury by inhibiting HMGB1

**DOI:** 10.1186/s13041-016-0260-0

**Published:** 2016-08-20

**Authors:** Jung Ho Lee, Eun Jang Yoon, Jeho Seo, Adriana Kavoussi, Yong Eun Chung, Sung Phil Chung, Incheol Park, Chul Hoon Kim, Je Sung You

**Affiliations:** 1 Department of Pharmacology, BK21 PLUS Project for Medical Science, Brain Research Institute, Yonsei University College of Medicine, 50-1 Yonsei-ro, Seodaemun-gu, Seoul 03722 South Korea; 2 Department of Radiology, Yonsei University College of Medicine, Seoul, 03722 South Korea; 3 Department of Emergency Medicine, Yonsei University College of Medicine, 50-1 Yonsei-ro, Seodaemun-gu, Seoul 03722 South Korea; 4 Severance Biomedical Science Institute, Yonsei University College of Medicine, Seoul, 03722 South Korea

**Keywords:** Acute ischemic stroke, High mobility group box 1 (HMGB1), Inflammatory cytokines, Penumbra, Hypothermia, Glycyrrhizin

## Abstract

Acute ischemic stroke causes significant chronic disability worldwide. We designed this study to clarify the mechanism by which hypothermia helps alleviate acute ischemic stroke. In a middle cerebral artery occlusion model (4 h ischemia without reperfusion), hypothermia effectively reduces mean infarct volume. Hypothermia also prevents neurons in the infarct area from releasing high mobility group box 1 (HMGB1), the most well-studied damage-associated molecular pattern protein. By preventing its release, hypothermia also prevents the typical middle cerebral artery occlusion-induced increase in serum HMGB1. We also found that both glycyrrhizin-mediated inhibition of HMGB1 and intracerebroventricular neutralizing antibody treatments before middle cerebral artery occlusion onset diminish infarct volume. This suggests a clear neuroprotective effect of HMGB1 inhibition by hypothermia in the brain. We next used real-time polymerase chain reaction to measure the levels of pro-inflammatory cytokines in peri-infarct regions. Although middle cerebral artery occlusion increases the expression of interleukin-1β and tissue necrosis factor-α, this elevation is suppressed by both hypothermia and glycyrrhizin treatment. We show that hypothermia reduces the production of inflammatory cytokines and helps salvage peri-infarct regions from the propagation of ischemic injury via HMGB1 blockade. In addition to suggesting a potential mechanism for hypothermia’s therapeutic effects, our results suggest HMGB1 modulation may lengthen the therapeutic window for stroke treatments.

**Electronic supplementary material:**

The online version of this article (doi:10.1186/s13041-016-0260-0) contains supplementary material, which is available to authorized users.

## Introduction

Traditional stroke treatments comprise aspirin, surgery, and thrombolysis [1–3]. If they can be rapidly applied, intravenous tissue plasminogen activator (IV-tPA) and intra-arterial thrombolysis are the most effective treatments for ischemic strokes [4]. Although it has long been recognized that treatment should begin within 3 ~ 4.5 h of stroke onset [3, 5], circumstances prevent many patients from receiving the most effective treatments within the optimal time interval [6, 7]. Thus, new adjunctive treatments that extend this critical therapeutic window will be invaluable in improving outcomes for patients with ischemic brain injuries.

The ischemic penumbra is an important target for stroke therapeutics. According to its original delineation, the ischemic penumbra is the region of brain tissue receiving reduced cerebral blood flow that surrounds the infarct core [8]. If a therapeutic intervention fails, the ischemic penumbra can also be encompassed into the developing infarct core [9, 10]. Therapeutic hypothermia has been suggested as a way to ‘freeze’ the ischemic penumbra and prolong the therapeutic window for acute ischemic stroke [11, 12]. Therapeutic hypothermia—accomplished by cooling a patient’s core body temperature to 32–34 °C for 12–24 h—offers strong neuroprotection for survivors of cardiac arrest [13]. Although the therapeutic efficacy of hypothermia for acute ischemic stroke has not yet been established in a large-scale clinical trial, several smaller studies have successfully demonstrated feasibility [14, 15]. In rodent models, hypothermia seems to reduce both ischemic core and penumbral injuries [16–19]. Hypothermia is known to reduce excitatory neurotransmitter release and free radical production, maintain blood brain barrier (BBB) integrity, and mitigate ischemia-induced inflammation [19–22]. Still, the principal molecular mediator of these neuroprotective effects of hypothermia is unknown.

Acute ischemic strokes trigger an innate immune response, leading to severe inflammation [23]. High mobility group box 1 (HMGB1), which is released from several types of cells upon injury, is one of the primary mediators of this innate immune response [24, 25]. HMGB1 is also a critical mediator of both the primary and secondary damage caused by ischemic strokes [24–26]. Indeed, interruption of HMGB1’s role as a damage-associated molecular pattern (DAMP) protein helps prevent the propagation of ischemic injury [24]. Extracellular HMGB1 binds to toll-like receptor 4 and receptor for advanced glycation end-products expressed in immune-competent cells, neurons, and astrocytes [27, 28]. HMGB1 binding acts as a danger signal, activating inflammatory mediators that then amplify and expand the extent of brain damage [26]. For example, in glial and endothelial cell, HMGB1 induces the expression of inflammatory mediators, tissue necrosis factor (TNF)-α and intercellular adhesion molecule (ICAM)-1 [29]. The upregulation of metalloproteinase (MMP)-9 by HMGB1 in neurons and astrocytes damages blood–brain barrier and expands brain damage [30]. In stroke patients, serum HMGB1 levels positively correlate with stroke severity [26, 31]. These reports suggest that HMGB1 is a valuable molecular target for new adjunctive stroke therapies.

Here we present evidence using a rat middle cerebral artery occlusion (MCAO) model of ischemic stroke that hypothermia inhibits infarct volume expansion by preventing HMGB1 release from post-ischemic neurons and diminishes subsequent inflammatory responses in the peri-infarct region. This study clarifies the mechanism by which hypothermia exerts its therapeutic effects and suggests that further interventions aimed at blocking the actions of HMGB1 will be valuable additions to stroke treatment regimens.

## Results

### Therapeutic hypothermia reduces infarct volume in post-ischemic brains

**Fig. 1:**
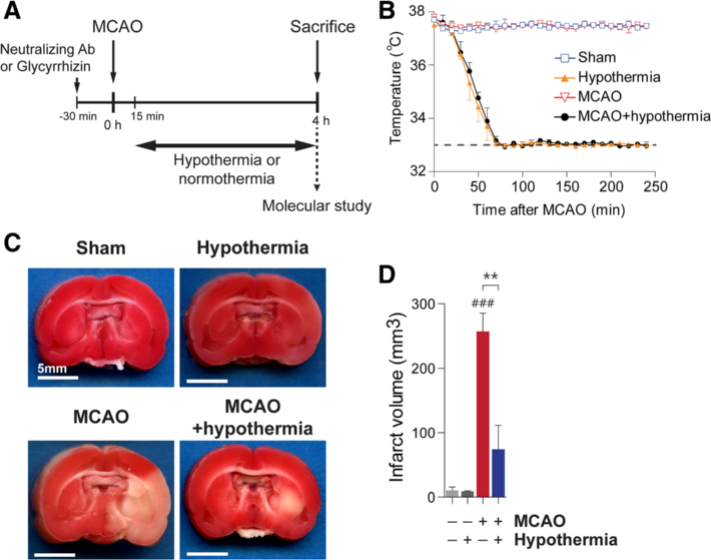
Hypothermia reduces ischemic infarct volume in MCAO rats. **a**, An illustration of the experimental schedule. **b**, Body temperature traces in rats after middle cerebral artery occlusion (MCAO). **c**, Representative images of 2,3,5-triphenyltetrazolium chloride (TTC) staining results. **d**, A quantification of infarct volume measured in TTC-stained brain slices of rats treated with MCAO and/or hypothermia. The number of rats in each group was as follows: sham (*n* = 3), hypothermia (*n* = 4), MCAO (*n* = 5), MCAO + hypothermia (*n* = 4). ### *P* < 0.001 versus sham alone, ** *P* < 0.01 comparing MCAO with and without hypothermia, one-way analysis of variance (ANOVA) followed by the Bonferroni *post hoc* test

In this study, we used a permanent MCAO model to explore the molecular changes induced by hypothermia prior to recanalization. We induced hypothermia 15 min after MCAO surgery and maintained it throughout the entire 4 h ischemic period (Fig. 1a). We ensured constant hypo- or normothermia by directly monitoring the core temperature of each rat. The core temperature of hypothermic rats fell to 33.0 ± 0.5 °C within 80 min and remained constant for 4 h following MCAO (Fig. 1b). The redox indicator 2,3,5-triphenyltetrazolium chloride (TTC) effectively delineates cerebral infarct volume with infarcted areas appearing devoid of red staining (Fig. 1c, see also in Additional file 1: Figure S1). Absent or attenuated staining of MAP2, a cytoskeletal protein that is highly sensitive to ischemic damage, also delineates a similar infarct area as the lack of TTC conversion to red-colored triphenylformazane does (in Additional file 2: Figure S2). Infarcts induced by 4 h MCAO are significantly diminished by hypothermia at 33 °C compared with normothermia (Fig. 1c, see also in Additional file 1: Figure S1). By measuring TTC staining-negative tissue volumes, we found that mild hypothermia at 33 °C attenuates mean infarct volume from 256.40 ± 29.01 mm^3^ to 73.60 ± 37.67 mm^3^ (Fig. 1d). MCAO-induced neurological deficits were evaluated using behavioral tests and found to be improved by hypothermia treatment (in Additional file 3: Figure S3). These data indicate hypothermia protects brain cells against cerebral ischemic damage.

### Therapeutic hypothermia inhibits extracellular release of HMGB1 from ischemic brain tissue

**Fig. 2:**
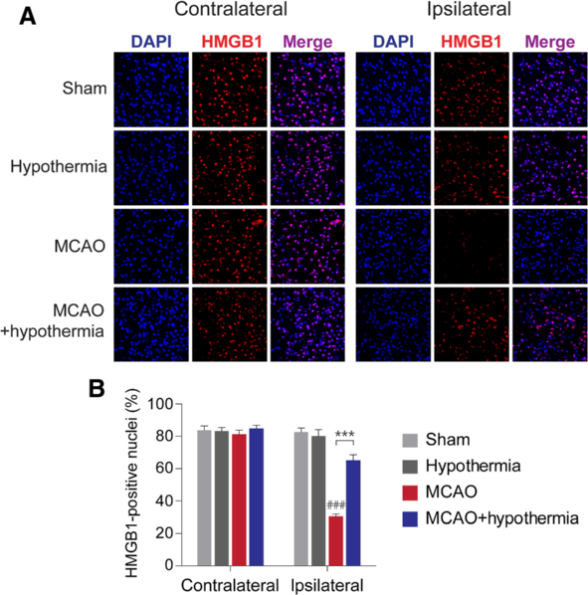
Hypothermia restores HMGB1 immunoreactivity in post-ischemic MCAO rat brains. **a**, Representative immunohistochemistry results from MCAO-treated rat brains in the absence or presence of hypothermia. **b**, A quantification of the immunohistochemistry results in **a**. The number of rats in each group was as follows: sham (*n* = 4), hypothermia (*n* = 4), MCAO (*n* = 6), MCAO + hypothermia (*n* = 4). ### *P* < 0.001 versus sham alone, *** *P* < 0.001 comparing MCAO with and without hypothermia, one-way analysis of variance (ANOVA) followed by the Bonferroni *post hoc* test

**Fig. 3:**
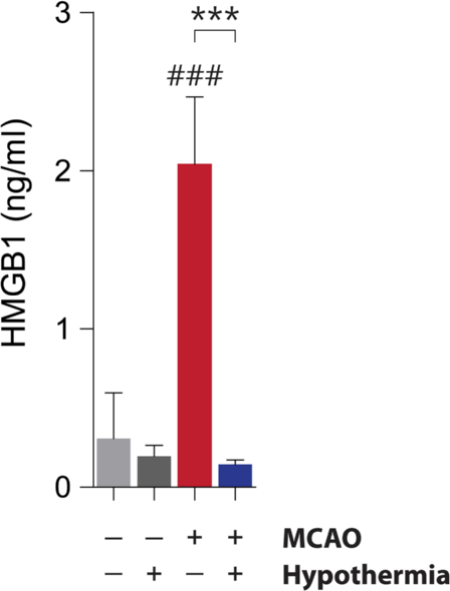
Hypothermia reduces serum HMGB1 levels in MCAO rats. ELISA assay for HMGB1 performed on sera drawn from rats 4 h after sham or MCAO surgery in the absence or presence of hypothermia. The number of rats in each group was as follows: sham (*n* = 4), hypothermia (*n* = 4), MCAO (*n* = 4), MCAO + hypothermia (*n* = 4). ### *P* < 0.001 versus sham alone, *** *P* < 0.001 comparing MCAO with and without hypothermia, one-way analysis of variance (ANOVA) followed by the Bonferroni *post hoc* test

**Fig. 4:**
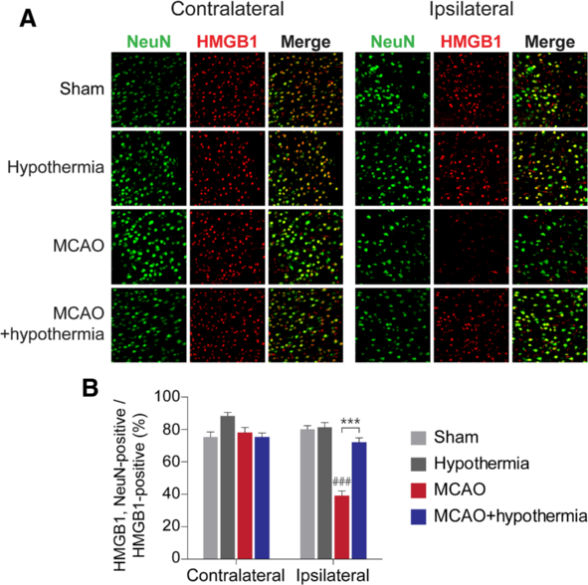
Hypothermia reverses the decrease in NeuN/HMGB1-double positive cells in MCAO rat brains. **a**, Representative immunohistochemistry results using antibodies against NeuN and HMGB1. **b**, A quantification of the immunohistochemistry results in **a**. The number of rats in each group was as follows: sham (*n* = 3), hypothermia (*n* = 3), MCAO (*n* = 5), MCAO + hypothermia (*n* = 3). ### *P* < 0.001 versus sham alone, *** *P* < 0.001 comparing MCAO with and without hypothermia, one-way analysis of variance (ANOVA) followed by the Bonferroni *post hoc* test

Upon MCAO-induced ischemic injury, HMGB1 is released from brain cell nuclei, reducing the number of HMGB1-positive cells in the ischemic cortex [24, 29]. We too found that HMGB1 immunoreactivity disappears from the cortex of MCAO rats, but that hypothermia significantly restores HMGB1 staining in the post-ischemic cortex. This suggests hypothermia attenuates the extracellular release of HMGB1 (Fig. 2a). We observed that while 30.31 ± 1.60 % of 4,6-diamidino-2-phenylindole (DAPI)-positive cells in the cortex of ischemic hemispheres (ipsilateral) were also HMGB1-postive, hypothermia increases this number roughly two-fold to 65.04 ± 3.53 % (Fig. 2b). Next, we performed an ELISA to measure HMGB1 levels in serum samples obtained 4 h after the onset of ischemia. As expected, the level of circulating of HMGB1 rises after MCAO, but its rise is significantly attenuated by hypothermia (Fig. 3). To evaluate if HMGB1 rise by MCAO affects neuron directly, we examined whether HMGB1 is preferentially depleted in the neuronal marker-positive cells. We stained rat brain sections with antibodies against HMGB1 and the neuronal marker NeuN. In the ipsilateral sham group, we found that 80.02 ± 2.27 % of HMGB1-positive cells were also NeuN-positive (Fig. 4a and b). Studies using the *in vivo* MCAO model and an oxygen-glucose deprivation *in vitro* culture model both report similar selective neuronal release of HMGB1 [26, 29]. In our MCAO rats, we observed a significant drop in the percentage of NeuN/HMGB1 double-positive cells to 39.0 ± 2.94 % of HMGB1-positive cells. This reduction, too, is dramatically restored by hypothermia—treated rats show 71.98 ± 2.72 % NeuN/HMGB1-double positive cells (Fig. 4a and b). These results indicate that hypothermia blocks the release of HMGB1 from ischemic rat neurons post-MCAO.

### Glycyrrhizin ameliorates MCAO-induced ischemic brain injury

**Fig. 5:**
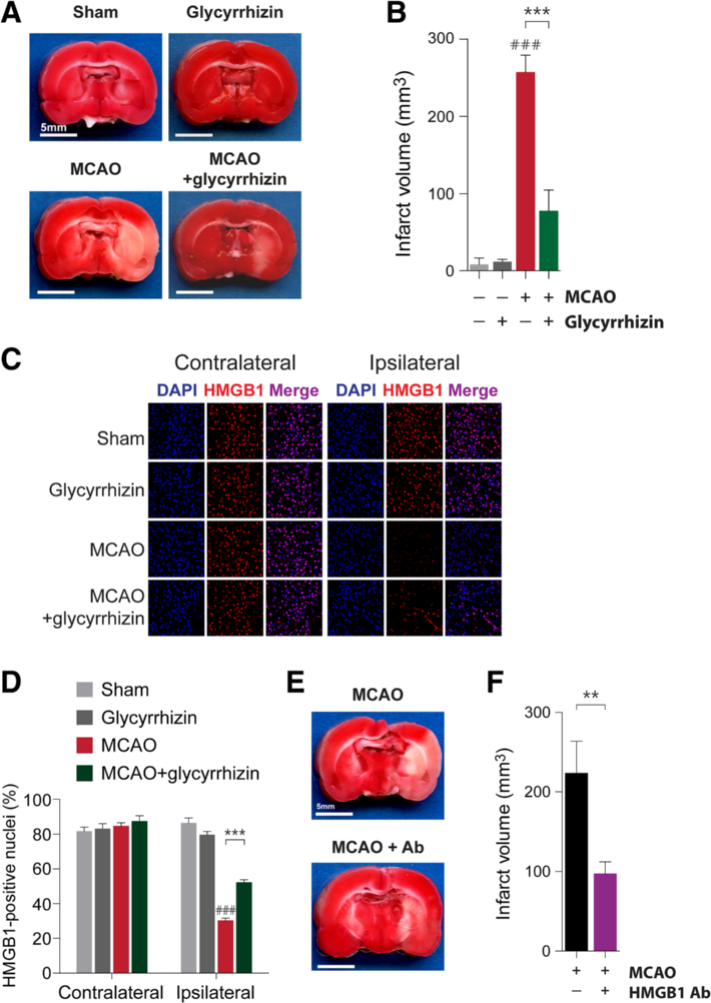
Glycyrrhizin and HMGB1 neutralizing antibodies reduce MCAO-induced ischemic brain injury. **a**, Representative TTC-staining results. A mixture of glycyrrhizin (100 mg/kg) and saline was administered intraperitoneally 30 min before MCAO. **b**, A quantification of the TTC staining results in **a**. The number of rats in each group was as follows: sham (*n* = 4), glycyrrhizin (*n* = 4), MCAO (*n* = 5), MCAO + glycyrrhizin (*n* = 5). ### *P* < 0.001 versus sham alone, *** *P* < 0.001 comparing MCAO with and without glycyrrhizin, one-way analysis of variance (ANOVA) followed by the Bonferroni *post hoc* test. **c**, Representative images showing HMGB1 immunoreactivity in brain sections from MCAO and/or glycyrrhizin-treated rats. **d**, A quantification of the immunohistochemistry results in **c**. The number of rats in each group was as follows: sham (*n* = 3), glycyrrhizin (*n* = 3), MCAO (*n* = 4), MCAO + glycyrrhizin (*n* = 4). ### *P* < 0.001 versus sham alone, *** *P* <0.001 comparing MCAO with and without glycyrrhizin, one-way ANOVA followed by the Bonferroni *post hoc* test. **e**. Representative images of TTC staining results. HMGB1 neutralizing antibodies (5 μg of antibody in 5 μl of PBS) were administered to the rats by intracerebroventricular injection 30 min before MCAO. **f**, A quantification of the TTC staining results in **e**. The number of rats in each group was as follows: MCAO (*n* = 3), MCAO + neutralizing antibody (*n* = 8). ** *P* < 0.01 comparing MCAO with and without the neutralizing antibody, unpaired *t*-test

Glycyrrhizin is a pharmacological HMGB1 inhibitor that has been suggested to bind directly to HMGB1 blocking its function as a cytokine [32] and to prevent cellular HMGB1 release [33, 34]. In an effort to verify that the neuroprotection conferred by hypothermia in our experimental MCAO model acts via inhibition of HMGB1, we measured the effect of glycyrrhizin treatment on infarct volume as well as HMGB1 release. Intra-peritoneal injection of glycyrrhizin markedly attenuates infarct volume in the post-ischemic cortex (257.20 ± 21.93 mm^3^ in MCAO rats versus 77.35 ± 27.19 mm^3^ in glycyrrhizin-treated MCAO rats, *P* < 0.001) (Fig. 5a and b, see also in Additional file 4: Figure S4). Glycyrrhizin also significantly increases the percentage of HMGB1-positive cells in the ischemic hemisphere (30.23 ± 1.34 % in MCAO rats versus 52.17 ± 1.59 % in glycyrrhizin-treated MCAO rats, *P* < 0.001) (Fig. 5c and d). Interestingly, glycyrrhizin inhibits HMGB1 release to levels similar to those we observed in MCAO rats treated with hypothermia. Together, these results suggest that the extracellular release of HMGB1 during an ischemic event is essential in the spread of ischemic injury and that therapeutic hypothermia prevents this spread via inhibition of HMGB1.

### Intracerebroventricular injection of HMGB1 neutralizing antibodies prevents ischemic brain injury

To determine the effect of a more specific inhibition of HMGB1 on ischemic injury, we injected 5 μg of a neutralizing antibody against HMGB1 into the intracerebroventricular space of rats. We injected the HMGB1 neutralizing antibody using an infusion pump over the course of 5 min beginning 30 min prior to the onset of ischemia. TTC staining after 4 h of ischemia showed that HMGB1 neutralizing antibody treatment reduces MCAO-induced cortical infarct volume (Fig. 5e and f, see also in Additional file 5: Figure S5). This result provides further evidence for our hypothesis that HMGB1 inhibition effectively protects the brain against the spread of ischemic injury.

### Both therapeutic hypothermia and glycyrrhizin inhibit inflammatory cytokine expression in peri-infarct regions

**Fig. 6:**
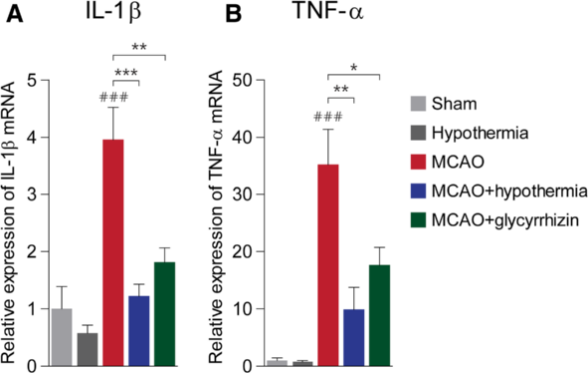
Pro-inflammatory cytokine expression in the peri-infarct region after MCAO. **a**, Quantification of interleukin-1β (IL-1β) expression by RT-PCR. ### *P* < 0.001 versus sham alone, ** *P* < 0.01, *** *P* < 0.001, one-way analysis of variance (ANOVA) followed by the Bonferroni *post hoc* test. **b**, Quantification of tissue necrosis factor-α (TNF-α) expression by RT-PCR. ### *P* < 0.001 versus sham alone, * *P* < 0.05, ** *P* < 0.01, one-way ANOVA followed by the Bonferroni *post hoc* test. The number of rats in each group was as follows: sham (*n* = 5), hypothermia (*n* = 5), MCAO (*n* = 8), MCAO + hypothermia (*n* = 6), MCAO + glycyrrhizin (*n* = 9)

Inflammatory cytokines released from the ischemic penumbra likely contribute to the extensive damage in the penumbra after an acute ischemic stroke [35–38]. To determine whether therapeutic hypothermia and glycyrrhizin alter the expression of inflammatory cytokines, we used RT-PCR to examine the mRNA levels of two major inflammatory cytokines (i.e., IL-1β and TNF-α) in the peri-infarct region 4 h after MCAO. We defined the peri-infarct region or penumbra as tissue within 2 mm of the infarct border. We decided to use the hindlimb region of the primary sensory cortex for our penumbral expression analysis because it consistently fell within the 2 mm range in all the brain slices we used for TTC staining and because it lies within a region showing perfusion-diffusion mismatch in a permanent MCAO rat model [39]. We found that although ischemic injury increases the expression of interleukin-1β (IL-1β) in the peri-infarct region, both hypothermia and glycyrrhizin treatments prevent this increase (Fig. 6a). Similarly, MCAO-induced ischemic injury increases peri-infarct tissue necrosis factor-α (TNF-α) expression, but both hypothermia and glycyrrhizin significantly reduce this increase (Fig. 6b). These results suggest hypothermia helps prevent infarct propagation by suppressing inflammatory cytokine production in peri-infarct regions via its inhibition of HMGB1.

## Discussion

In rodent models of stroke, therapeutic hypothermia reportedly reduces infarct volume and post-ischemic inflammation, salvaging much of the ischemic penumbra [16, 18, 19, 21, 40]. Previously, Koda et al. reported that hypothermia reduces HMGB1 in rat cortical lysates after bilateral common carotid artery (CCA) occlusion and hypotension [41]. Although the ischemic models (i.e., bilateral CCA occlusion with hypotension vs. MCAO) and duration of ischemic injury (i.e., 120 min reperfusion after 10 min ischemia vs. 4 h MCAO in our study) were different, both studies support the intriguing new hypothesis that hypothermia acts to inhibit HMGB1 in acute ischemic injury. This idea is even more clinically relevant in light of a recent study showing that serum HMGB1 levels may be a valuable prognostic marker in stroke patients [31]. It remains unclear, however, whether hypothermia suppresses HMGB1 action at the site of injury, as well as in adjacent and distant tissues *in vivo*. It is also unclear how HMGB1 participates in ischemic injury propagation and exactly what the consequences of its suppression may be. Our current study is the first direct functional and mechanistic link between HMGB1 and therapeutic hypothermia in a clinically relevant permanent MCAO animal model.

We have also shown that the HMGB1 inhibitor glycyrrhizin attenuates pro-inflammatory cytokines in MCAO rat brains just like therapeutic hypothermia does. This suggests hypothermia’s inhibition of HMGB1 accounts for its suppression of peri-infarct inflammation. IL-1β and TNF-α dramatically affect infarct evolution in experimental stroke models [38, 42, 43]. Indeed, neutralization of IL-1β and TNF-α reduces infarct size in the MCAO model [44, 45]. Cytokines are produced either in the peri-infarct region or in numerous mini-penumbras inside the infarct core, and both sources likely promote the propagation of ischemic damage [46, 47]. According to some reports, IL-1β and TNF-α expression begin to increase between 4 and 6 h after the onset of ischemia [38, 46, 48]. In the permanent MCAO model, though, a substantial fraction of the penumbra is recruited into the infarct core even 1 h after the onset of ischemia [38, 49]. This makes it seem unlikely that these cytokines and the HMGB1 that induces them are important in the evolution of a reversible episode of ischemia to an area of irreversible damage. In our real-time PCR experiments, though, we already observed robust increases of both IL-1β and TNF-α expression at 4 h of permanent MCAO induced ischemia. The fact that other studies generally measure changes in cytokine expression in whole brain lysates rather than a defined peri-infarct region [46, 48] may hinder their ability to observe early and highly localized mRNA changes. Our new data from the peri-infarct region suggest the involvement of pro-inflammatory cytokines in infarct development and ischemic injury propagation much earlier than previously thought. Hypothermia seems to interfere with these processes via its inhibition of HMGB1. Our results also suggest that HMGB1 inhibitors are worth being assessed for their ability to act as neuroprotectants in human ischemic stroke. Glycyrrhizin has been used to treat chronic hepatitis C infection [50]. However, it necessitates more pre-clinical and clinical studies to prove its efficacy and safety in ischemic stroke patients.

One advantage of hypothermia is that it may protect against ischemic injury even if it is applied after the ischemic insult [17]. Koda et al. reported that hypothermia induced after an ischemic event fails to reduce serum HMGB1 levels in rats [41], suggesting that HMGB1 is irrelevant for hypothermia’s protective effects. In contrast, we found that induction of hypothermia 15 min after an ischemic insult effectively reduces both serum HMGB1 and infarct volume. Consistent with our results, Liu et al. found that post-ischemic administration of a neutralizing antibody against HMGB1 protects against ischemic brain injury [51]. This implies that the effectiveness of post-ischemic hypothermia is indeed related to its inhibition of HMGB1. We expect that further research into the relationships between the time of hypothermia application, the extent of HMGB1 release, and the degree of therapeutic effect will be enormously beneficial.

This study provides evidence that therapeutic hypothermia inhibits the propagation of ischemic brain damage by inhibiting the extracellular release of HMGB1.

## Methods

### Animal preparation

Healthy male Wistar rats weighing 295–315 g were used for all experiments. All animal experiments were performed in compliance with guidelines approved by the Institutional Animal Care and Use Committee (IACUC) of Yonsei University Health System and according to National Institutes of Health guidelines.

### Experimental MCAO model

Focal brain ischemia was induced via intraluminal suturing of the middle cerebral artery [52]. Anesthesia was induced with 5 % isoflurane in a mixture of 0.7 L/min nitrous oxide and 0.3 L/min oxygen and maintained using 2 % isoflurane in the same gas mixture. The external carotid artery (ECA) was ligated and coagulated after isolating it and its branches. The internal carotid artery (ICA) was also carefully isolated from the adjacent vagus nerve. After ligation of the pterygopalatine artery, the common carotid artery (CCA) was also ligated. Next, the proximal ICA was loosely tied with a 6–0 black silk suture, and a microvascular clip was applied across the distal ICA. After making an incision in the proximal ICA, an intraluminal 4–0 MCAO suture (403556PK10, Doccol corporation, Sharon, MA) was inserted and the loosely tied 6–0 black silk suture on the proximal portion of ICA was tightened. After removing the clip, an intraluminal 4–0 MCAO suture was advanced from the proximal ICA lumen in the distal direction to a point approximately 22 mm beyond the CCA bifurcation. For drug treatment, glycyrrhizin (100 mg/kg) was injected intraperitoneally 30 min before the onset of MCAO.

### Temperature management

Animals were randomly divided into four groups: sham, hypothermia, MCAO, and MCAO + hypothermia. In the hypothermia group, surface cooling was begun 15 min after ischemic induction by placing ice packs on the rat torso. Vecuronium (0.9 mg/kg) was injected intramuscularly to inhibit shivering. After the sham and MCAO surgeries, the target core temperatures were carefully monitored and maintained for 4 h using a feedback-controlled heating pad (HB 101, Harvard apparatus, Holliston, MA) and surface cooling with ice packs.

### Infarct volume measurement

Rats were decapitated under anesthesia 4 h after sham or MCAO surgery. Coronal sections (2 mm-thick) were stained with 1 % TTC (T8877, Sigma-Aldrich, St. Louis, MO) solution at 37 °C for 10 min. After fixation, caudal and rostral faces of each section was scanned with a flatbed scanner. Scanned images were analyzed with ImageJ 1.48v (NIH, Bethesda, MD). Infarcted volumes (mm^3^) were calculated by multiplying the total averaged infarcted area by the section thickness; Thickness x (caudal area + rostral area)/2.

### Enzyme-linked immunosorbent assay (ELISA)

One ml of blood from the right atrium was withdrawn using a 23-gauge needle into a serum separator tube (BD, Plymouth, UK), centrifuged for 20 min at 2,000 rpm. HMGB1 concentrations were determined using the HMGB1 ELISA kit (ST51011, IBL International GmbH, Hamburg, Germany).

### Immunofluorescence

Two mm-thick rat brain slices (bregma 0.7 mm to −1.3 mm) were immersed in a 4 % paraformaldehyde solution and then cryoprotected with 30 % sucrose in phosphate-buffered saline (PBS). Sections with a thickness of 20 μm from 0.2 mm to −0.3 mm relative to the bregma were chosen for staining. Sections were first permeabilized and then incubated overnight at 4 °C with an anti-HMGB1 antibody (1:100, ab18256; Abcam, Cambridge, UK) or an anti-NeuN antibody (1:100, MAB377; Millipore, Billerica, MA). After washing, fluorescent dye-conjugated secondary antibodies were applied. Stained sections were observed under a LSM700 confocal microscope (Carl Zeiss, Jena, Germany).

### MAP-2 (microtubule associated protein-2) staining

Two mm-thick brain slices (bregma −1.3 mm to −3.3 mm) were immersed in a 4 % paraformaldehyde solution and then embedded in paraffin. Paraffin sectioning (4 μm-thick) was performed for immunohistochemistry. After deparaffinization, sections were incubated with an anti-MAP-2 antibody (1:100, M1406, Sigma) at 4 °C. After incubation with HRP-conjugated secondary antibodies, sections were stained with diaminobenzidine (K-3468, Dako). Images of sections were obtained using a motorized microscope (BX61VS, Olympus).

### Neurobehavioral testing

Hypothermic rats were passively rewarmed to 37 °C for 1 h after 3 h MCAO and were subjected to neurobehavioral tests. A modified Garcia 18-point scoring system was used for the evaluation of neurological deficits [53]. Six sensorimotor tests (spontaneous activity, symmetry in the movement of four limbs, forepaw outstretching, climbing, body proprioception, response to vibrissae touch; each scored on a scale of 0 ~ 3 or 1 ~ 3) were performed and the scores given to each rat were summated to derive total neurological deficit score (the maximum score 18, namely, healthy rats and the minimum score 3).

### Real-time polymerase chain reaction (RT-PCR)

Tissue RNA was extracted using the Hybrid-R kit (305–010, GeneAll biotechnology, Seoul, Korea). cDNAs were prepared from 1 μg of total RNA using the PrimeScript 1st strand cDNA Synthesis Kit (Takara Bio, Shiga, Japan). PCR amplification was performed using the SYBR-Green reagent (Takara Bio).

### Statistical analysis

All data are presented as means ± s.e.m. Differences between groups were analyzed using two-tailed unpaired *t* tests or one-way analysis of variance (ANOVAs) followed by Bonferroni *post hoc* tests for multiple comparisons between groups. *P* < 0.05 was considered significant.

## Additional files

Additional file 1: Figure S1. Representative image of TTC-stained serial coronal brain sections from MCAO-treated rats. (DOCX 1229 kb) Additional file 2: Figure S2. Comparison of infarct volume between TTC- and MAP-2-stained coronal brain sections. (DOCX 872 kb) Additional file 3: Figure S3. Neurobehavioral tests that assess MCAO-induced functional neurological deficits. (DOCX 34 kb) Additional file 4: Figure S4. Representative image of TTC-stained serial coronal brain sections from MCAO and/or glycyrrhizin-treated rats. (DOCX 1213 kb) Additional file 5: Figure S5. Representative image of TTC-stained serial coronal brain sections from MCAO and/or anti-HMGB1 antibodies-treated rats. (DOCX 1204 kb)

## Abbreviations

BBB: Blood brain barrier
CCA: Common carotid artery
DAMP: Damage-associated molecular pattern
DAPI: 4,6-diamidino-2-phenylindole
ECA: External carotid artery
ELISA: Enzyme-linked immunosorbent assay
HMGB1: High mobility group box 1
ICA: Internal carotid artery
IL-1β: Interleukin-1β
IV-tPA: Intravenous tissue plasminogen activator
MCAO: Middle cerebral artery occlusion
RAGE: Receptor for advanced glycation end-products
TLR4: Toll-like receptor 4
TNF-α: Tissue necrosis factor-α
TTC: 2,3,5-triphenyltetrazolium chloride
