# Supplementary material for: Hypothermia inhibits the propagation of acute ischemic injury by inhibiting HMGB1
Source: Mol Brain. 2016 Aug 20;9:81. doi: 10.1186/s13041-016-0260-0 (PMC4992290; doi:10.1186/s13041-016-0260-0)
Supplement: Additional file 1: Figure S1. — Representative image of TTC-stained serial coronal brain sections from MCAO-treated rats. (DOCX 1229 kb) [file 13041_2016_260_MOESM1_ESM.docx]

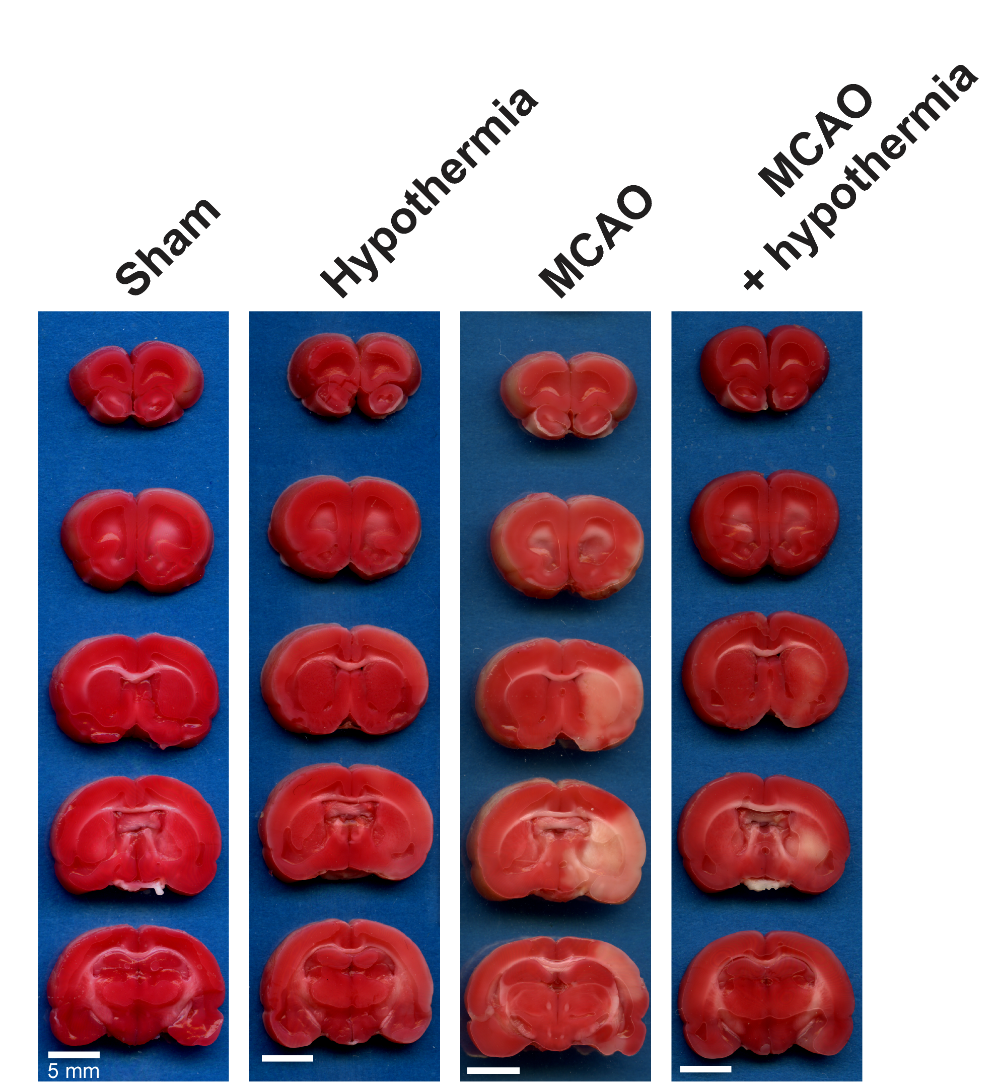


Figure S1. Representative images of TTC-stained serial coronal brain sections from MCAO-treated rats
