# Supplementary material for: Hypothermia inhibits the propagation of acute ischemic injury by inhibiting HMGB1
Source: Mol Brain. 2016 Aug 20;9:81. doi: 10.1186/s13041-016-0260-0 (PMC4992290; doi:10.1186/s13041-016-0260-0)
Supplement: Additional file 2: Figure S2. — Comparison of infarct volume between TTC- and MAP-2-stained coronal brain sections. (DOCX 872 kb) [file 13041_2016_260_MOESM2_ESM.docx]

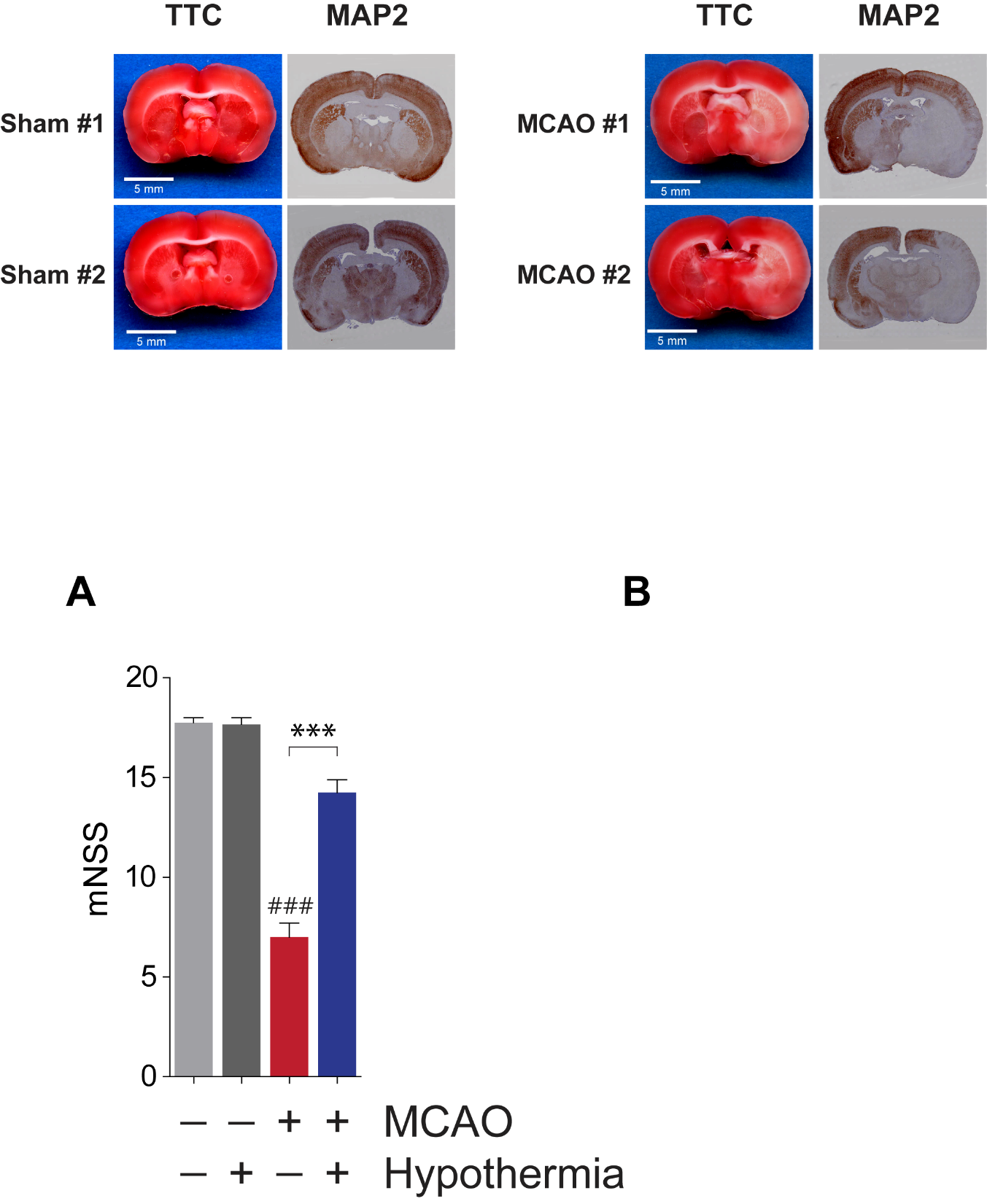


Figure S2. Comparison of infarct volume between TTC- and MAP-2-stained coronal brain sections. TTC-stained sections at bregma -1.3 ± 0.5 cm and MAP-2 stained brain sections at a similar coronal coordinate (0.3 ~ 0.5 mm posterior to the TTC-stained sections) from sham-treated and MCAO-treated rats.
