# Supplementary material for: Hypothermia inhibits the propagation of acute ischemic injury by inhibiting HMGB1
Source: Mol Brain. 2016 Aug 20;9:81. doi: 10.1186/s13041-016-0260-0 (PMC4992290; doi:10.1186/s13041-016-0260-0)
Supplement: Additional file 3: Figure S3. — Neurobehavioral tests that assess MCAO-induced functional neurological deficits. (DOCX 34 kb) [file 13041_2016_260_MOESM3_ESM.docx]

Figure S3. Neurobehavioral tests that assess MCAO-induced functional neurological deficits. Modified Garcia 18-point scoring system was used for the evaluation of neurological deficits with the maximum score 18 and the minimum score 3. The score in normal control rats would be 18. ### *P* < 0.001 versus sham alone, *** *P* < 0.001, MCAO versus MCAO + hypothermia group, one-way analysis of variance (ANOVA) followed by the Bonferroni *post hoc* test. The number of rats in each group was as follows: sham (n = 3), hypothermia (n = 3), MCAO (n = 4), MCAO + hypothermia (n = 4).
