# Supplementary material for: Hypothermia inhibits the propagation of acute ischemic injury by inhibiting HMGB1
Source: Mol Brain. 2016 Aug 20;9:81. doi: 10.1186/s13041-016-0260-0 (PMC4992290; doi:10.1186/s13041-016-0260-0)
Supplement: Additional file 5: Figure S5. — Representative image of TTC-stained serial coronal brain sections from MCAO and/or anti-HMGB1 antibodies-treated rats. (DOCX 1204 kb) [file 13041_2016_260_MOESM5_ESM.docx]

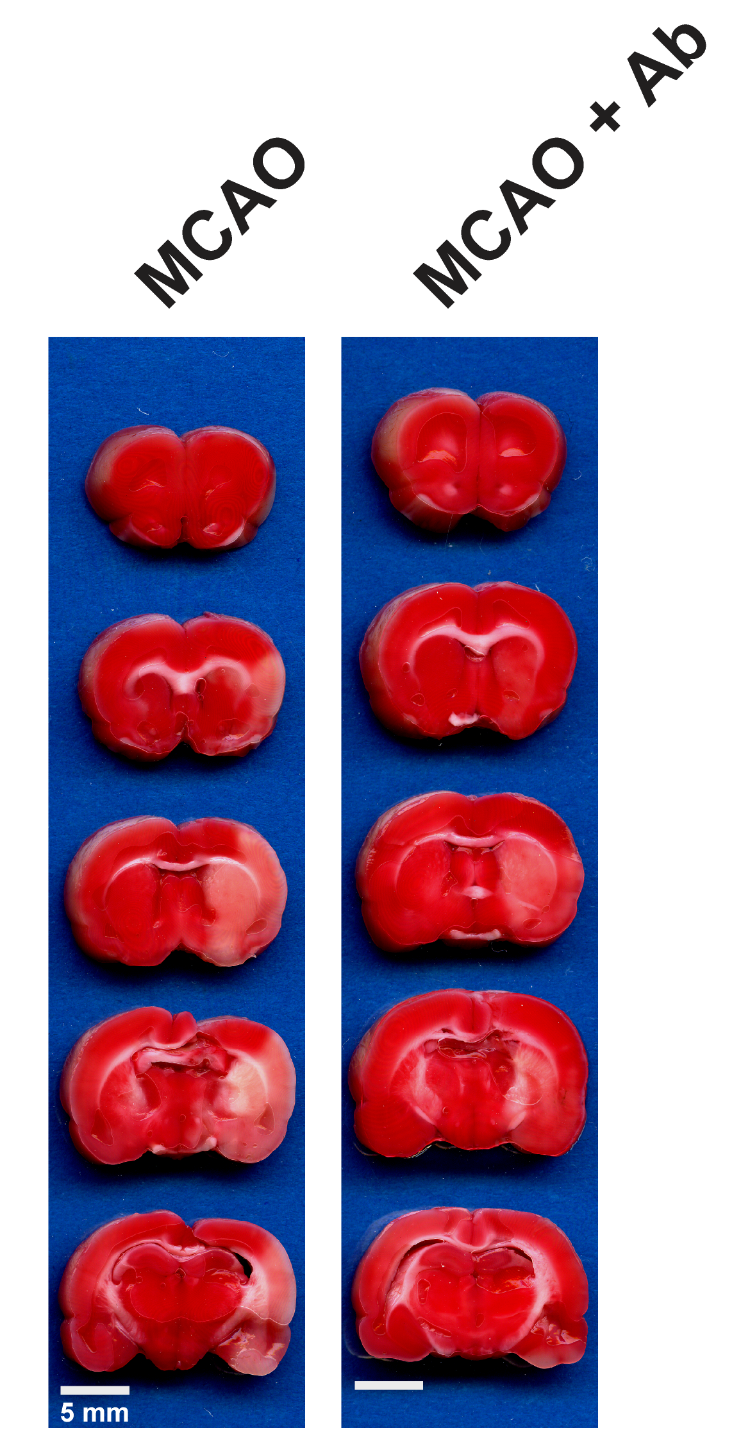


Figure S5. Representative image of TTC-stained serial coronal brain sections from MCAO and/or anti-HMGB1 antibodies-treated rats.
